# Supplementary material for: A disease resistance protein triggers oligomerization of its NLR helper into a hexameric resistosome to mediate innate immunity
Source: Sci Adv. 2024 Nov 6;10(45):eadr2594. doi: 10.1126/sciadv.adr2594 (PMC11540030; doi:10.1126/sciadv.adr2594)
Supplement: Supplementary file 1 — Figs. S1 to S18 Table S1 Legend for movie S1 Legends for data S1 and S2 [file sciadv.adr2594_sm.pdf]

Science Advances
AAAS

Supplementary Materials for

A disease resistance protein triggers oligomerization of its NLR helper into a hexameric resistosome to mediate innate immunity

Jogi Madhuprakash *et al.*

Corresponding author: Jogi Madhuprakash, Madhu.Jogi@tsl.ac.uk;
Michael W. Webster, Michael.Webster@jic.ac.uk; Sophien Kamoun, Sophien.Kamoun@tsl.ac.uk

Sci. Adv. **10**, eadr2594 (2024)
DOI: 10.1126/sciadv.adr2594

The PDF file includes:

Figs. S1 to S18
Table S1
Legend for movie S1
Legends for data S1 and S2

Other Supplementary Material for this manuscript includes the following:

Movie S1
Data S1 and S2

Fig. S1

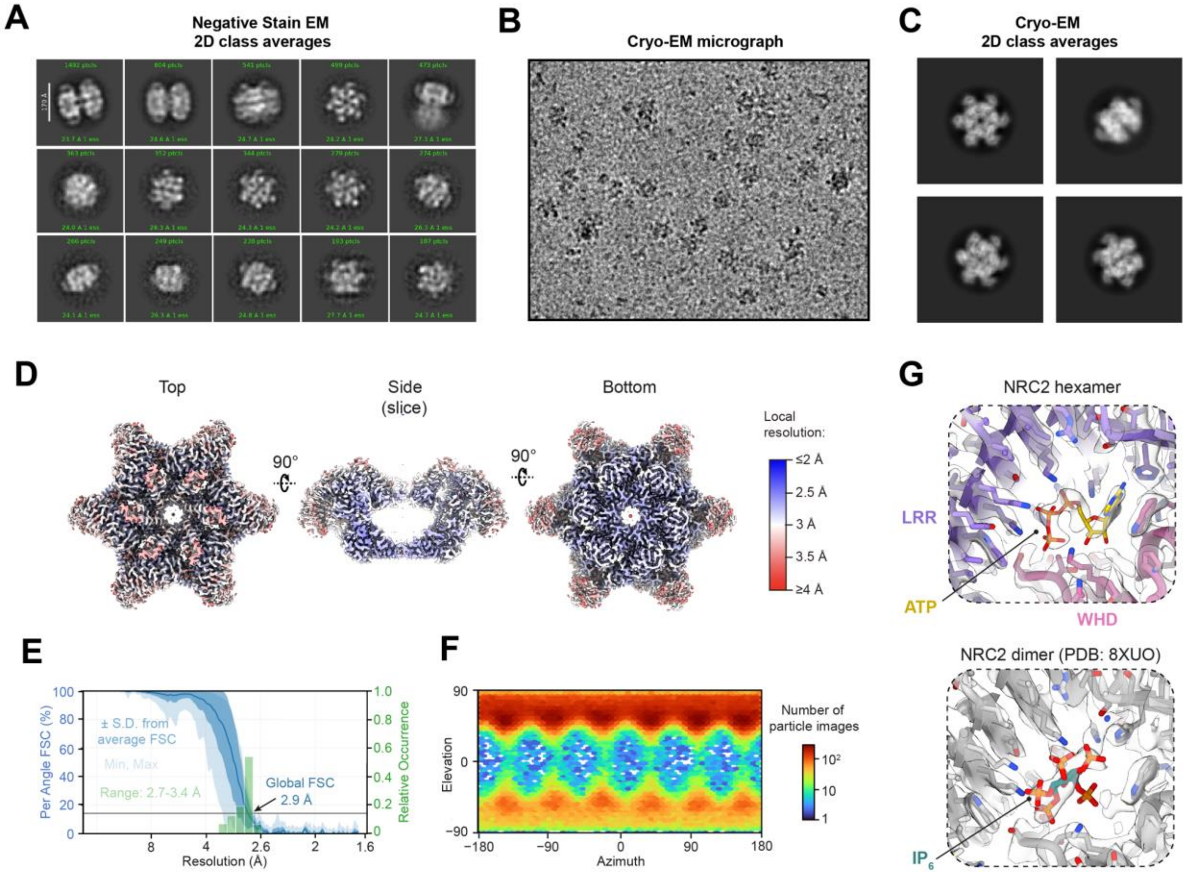

Fig. S1: Cryo-EM analysis reveals homohexameric resistosome structure of NbNRC2 activated with Rx and *Potato virus X* coat protein. (**A**) Analysis of purified NbNRC2^{EEE} by single-particle negative-stain electron microscopy and the longest dimension in the 3D reconstruction is 17 nm as indicated. (**B**) Representative cryo-EM micrograph of NbNRC2^{EEE}. (**C**) Selected cryo-EM 2D class averages from the final particle set. (**D**) Local resolution estimate for consensus NbNRC2^{EEE} resistosome after reconstruction. (**E**) Fourier shell correlation plot for consensus NbNRC2^{EEE} resistosome reconstruction. (**F**) Angular distribution plot for consensus NbNRC2^{EEE} resistosome reconstruction. (**G**) Cryo-EM reconstruction of the NRC2 hexamer (top) shows additional density in the concave surface of the LRR repeat consistent with a nucleotide triphosphate. The corresponding position in NRC2 dimer reconstruction (bottom, PDB: 8XUO) contained density at the corresponding position with a different shape that was identified as IP_6.

Fig. S2

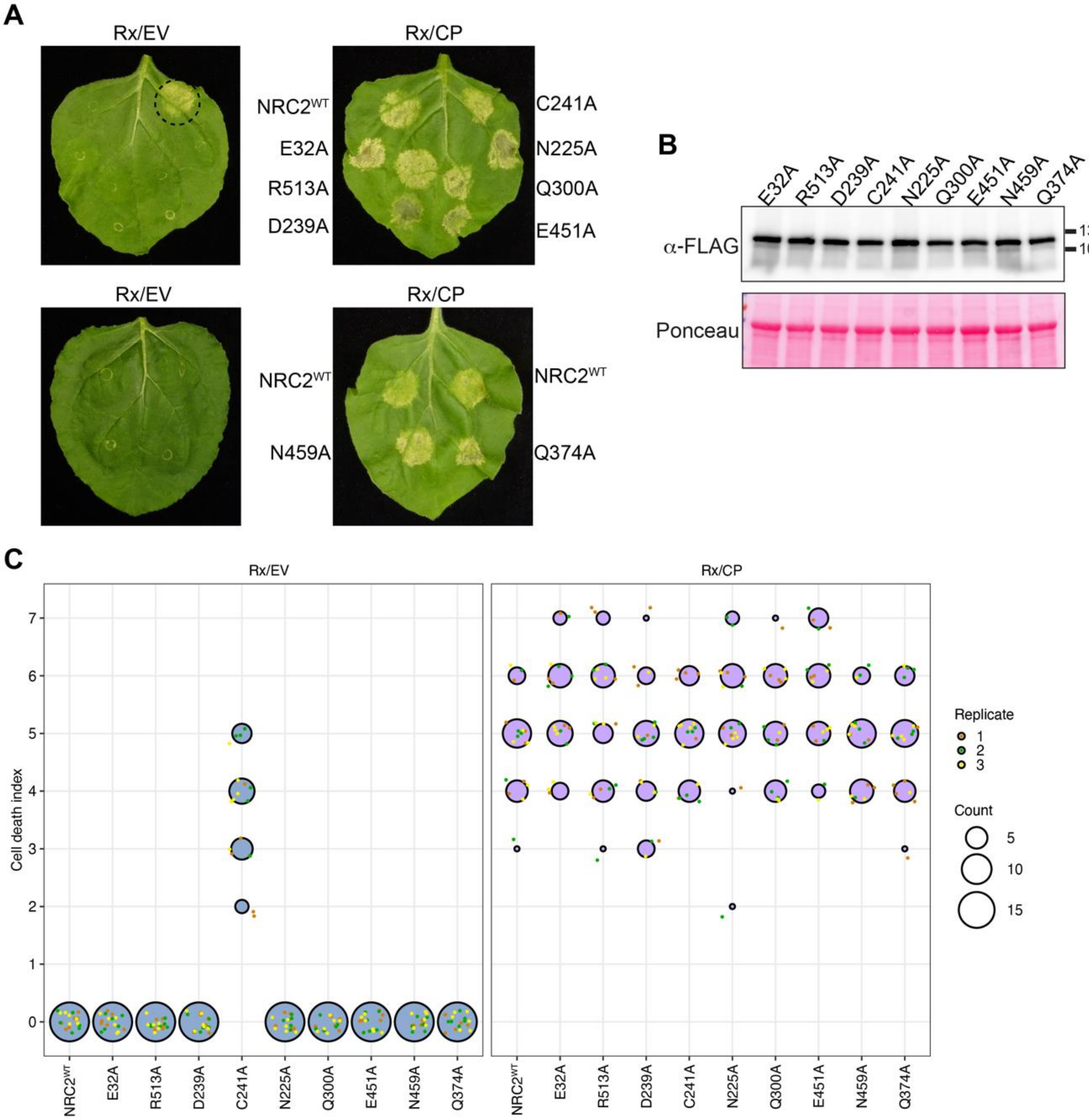

Fig. S2: NbNRC2 single amino acid mutants in the oligomerization interface trigger cell death upon activation by Rx/CP. (**A**) Photo of representative leaves from *N. benthamiana nrc2/3/4* KO plants showing HR after co-expression of NbNRC2 variants and Rx, together with either EV or CP. **(B)** SDS-PAGE of all NbNRC2 variants tested. Total protein extracts were immunoblotted with the appropriate antisera labelled on the left. Approximate molecular weights (kDa) of the proteins are shown on the right. Rubisco loading control was carried out using Ponceau stain (PS). The experiment was repeated three times with similar results. (**C**) HR scores accompanying panel (**A**). HR was scored based on a modified 0-7 scale between 5-

7 days post-infiltration. HR scores are presented as dot plots, where the size of each dot is proportional to the number of samples with the same score (Count). Results are based on 3 biological replicates.

Fig. S3

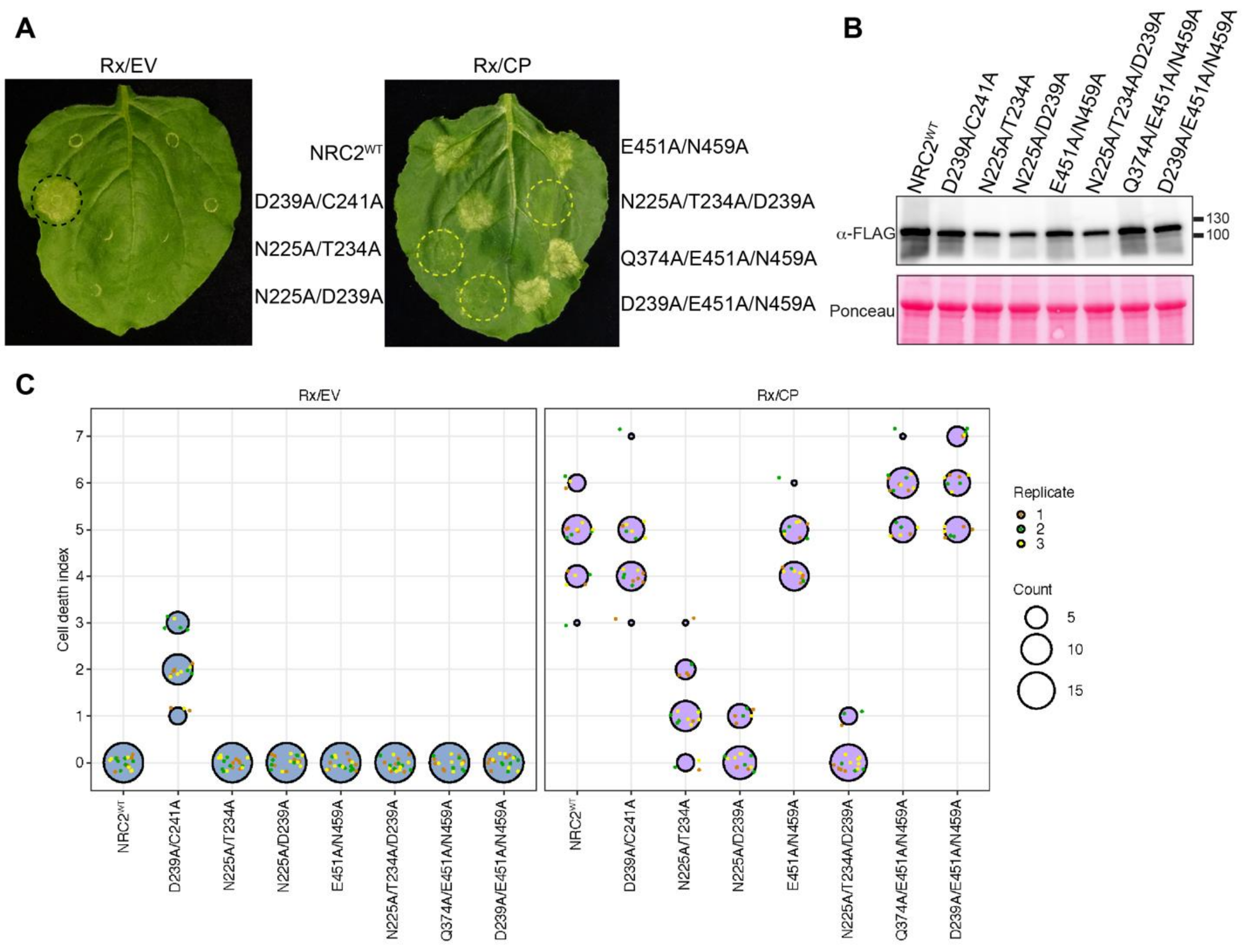

Fig. S3: Mutations in the NB-NB inter-protomer interface abolish NbNRC2-mediated cell death. (**A**) Photo of representative leaves from *N. benthamiana nrc2/3/4* KO plants showing HR after co-expression of NbNRC2 variants and Rx, together with either EV or CP. **(B)** SDS-PAGE of all NbNRC2 variants tested. Total protein extracts were immunoblotted with the appropriate antisera labelled on the left. Approximate molecular weights (kDa) of the proteins are shown on the right. Rubisco loading control was carried out using Ponceau stain (PS). The experiment was repeated three times with similar results. (**C**) HR scores accompanying panel (**A**). HR was scored based on a modified 0-7 scale between 5-7 days post-infiltration. HR scores are presented as dot plots, where the size of each dot is proportional to the number of samples with the same score (Count). Results are based on 3 biological replicates.

Fig. S4

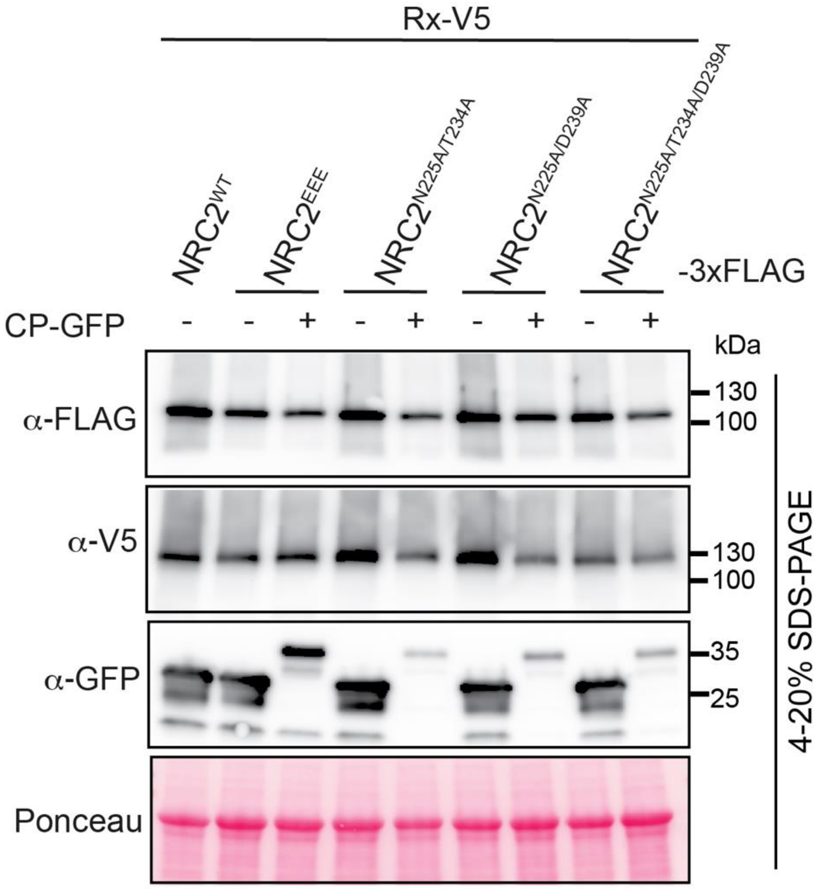

Fig. S4: Mutants in NbNRC2 inter-protomer oligomerization interface fail to form resistosomes. SDS-PAGE of all NbNRC2 variants tested, accompanying BN-PAGE experiment in **Fig. 2**. Total protein extracts were immunoblotted with the appropriate antisera labelled on the left. Free GFP was used as a control for CP-GFP. Approximate molecular weights (in kDa) of the proteins are shown on the right. Rubisco loading control was carried out using Ponceau stain (PS). The experiment was repeated three times with similar results.

Fig. S5

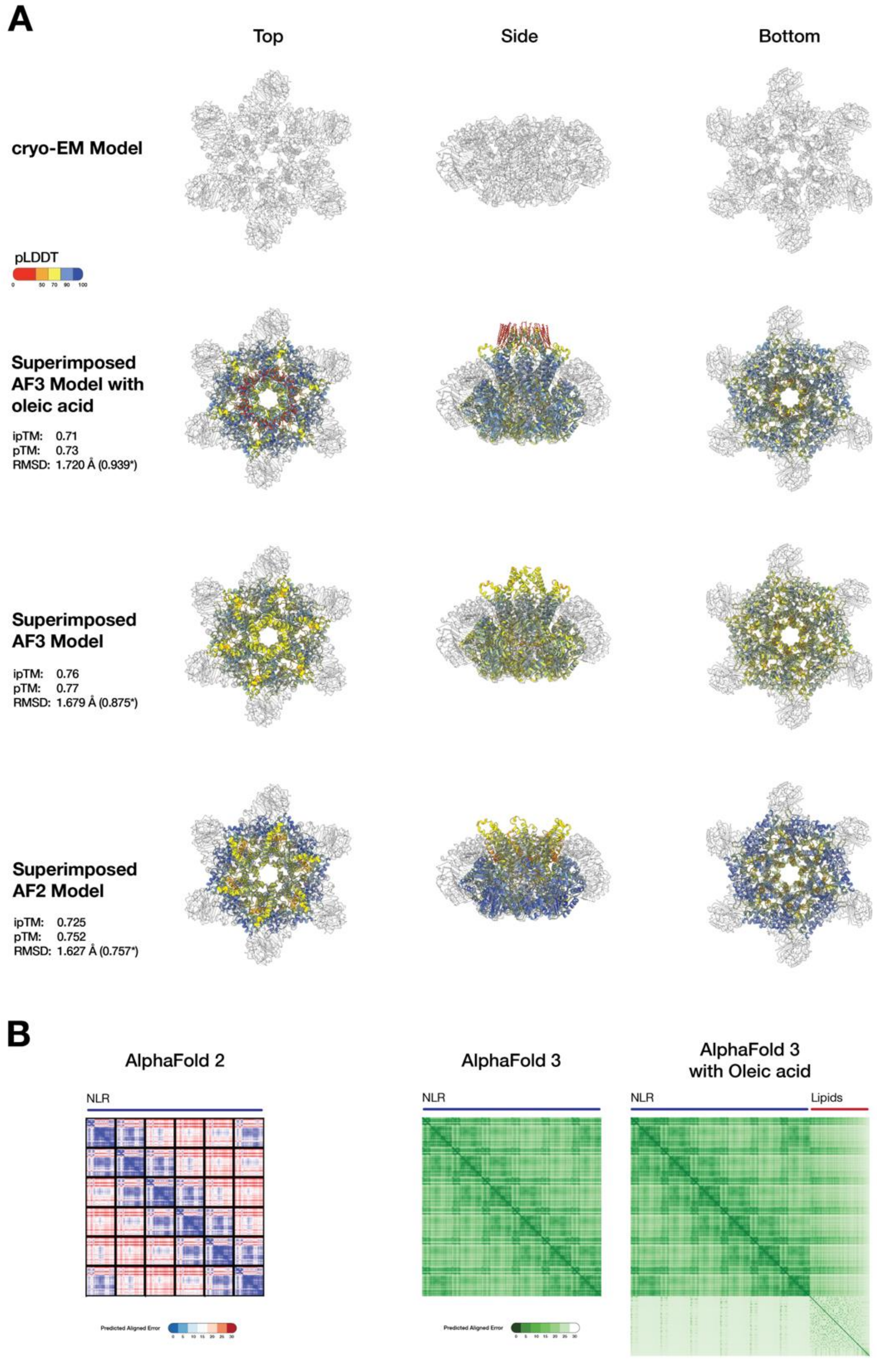

Fig. S5. AlphaFold 3 can predict a high-confidence NbNRC2 resistosome bound to the complete funnel structure. (**A**) Superimposition of NbNRC2 predicted structures from AlphaFold 3 with and without oleic acids, and AlphaFold 2 on the cryo-EM model. AlphaFold 3 predicted structure with oleic acid had a complete and confident lipid-bound funnel structure (**B**) Predicted aligned error plots for the three models from AlphaFold 2, AlphaFold 3, and AlphaFold 3 with oleic acid.

Fig. S6

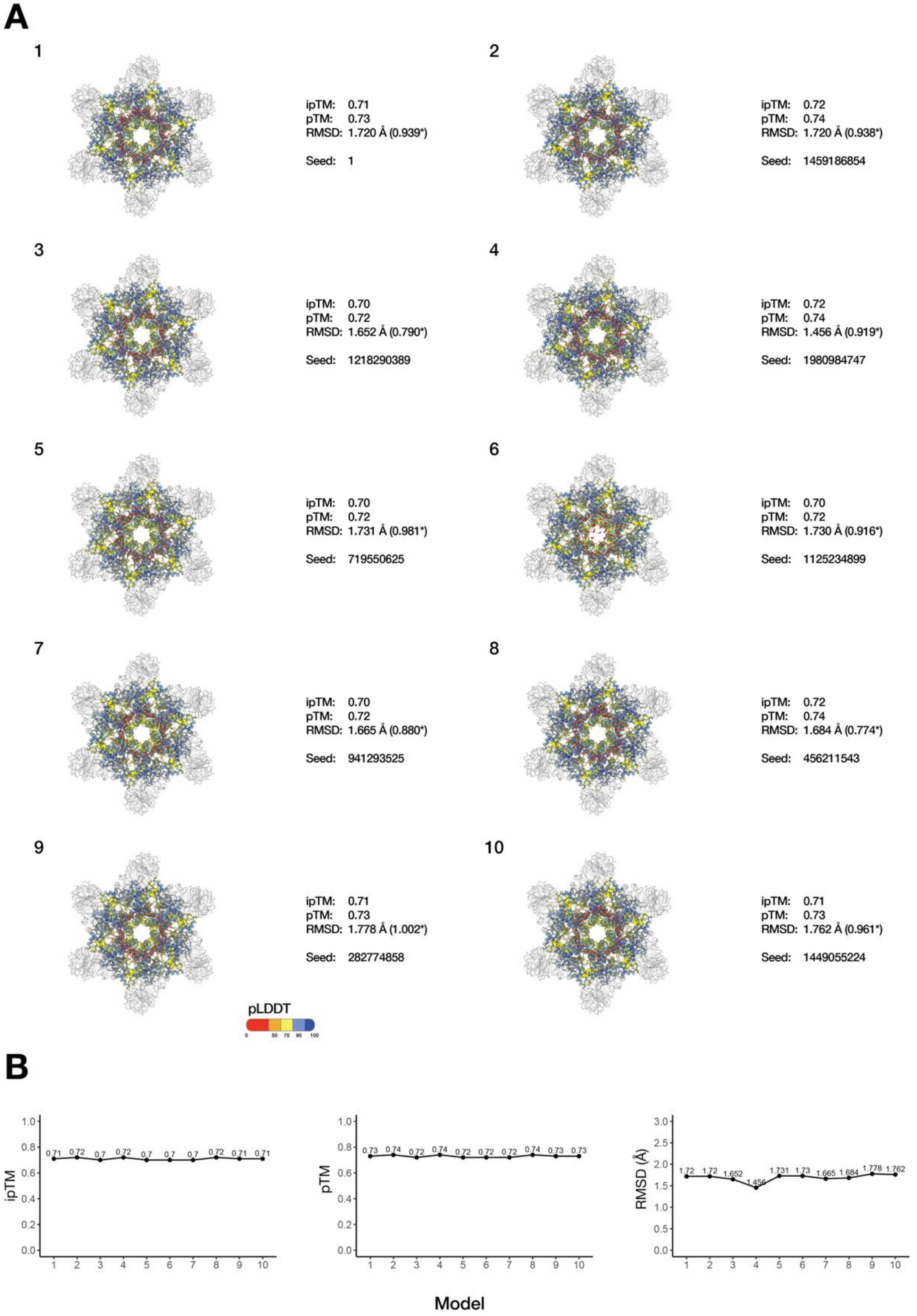

Fig. S6: AlphaFold 3 consistently produces high-confidence NbNRC2 resistosome models. (**A**) and (**B**) 10 generated NbNRC2 resistosomes with random seeds. All models aligned with NbNRC2 cryo-EM structure with RMSDs of 1.8 Å and lower.

Fig. S7

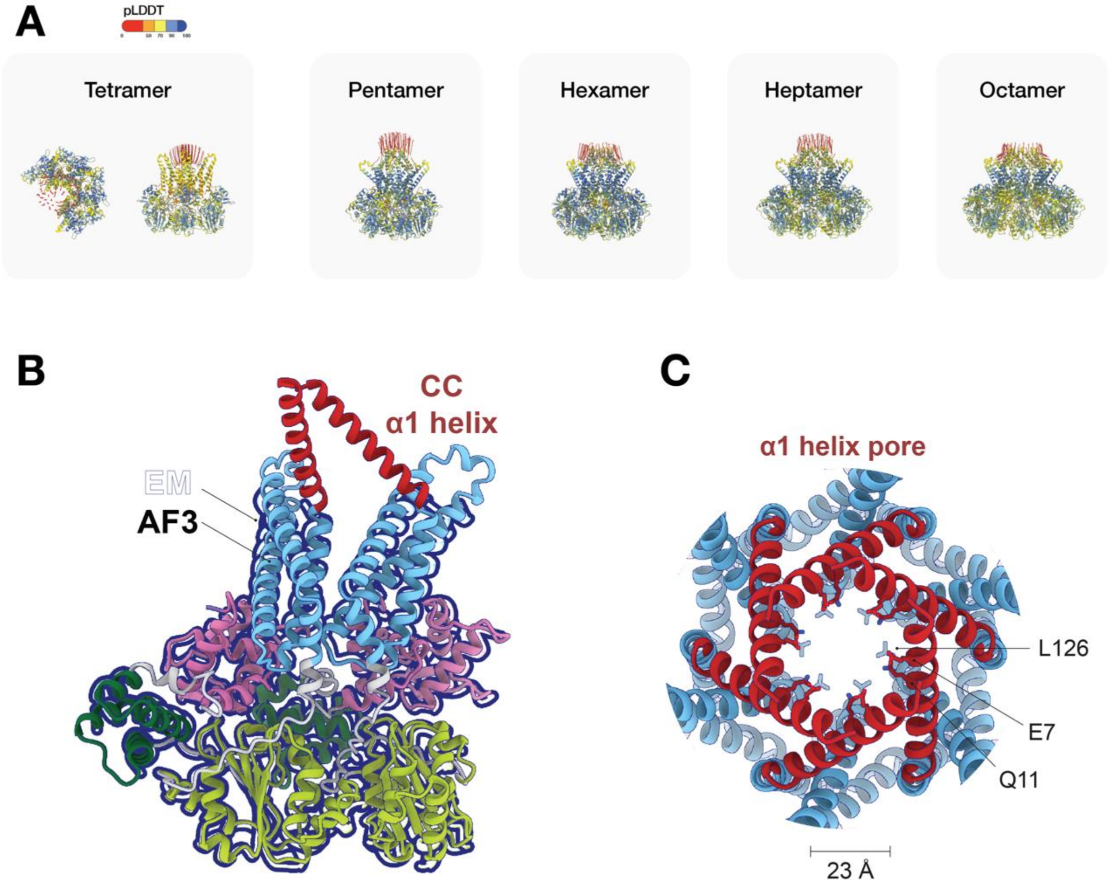

Fig. S7. AlphaFold 3 can predict different oligomeric configurations of NbNRC2 with variable confidences. (**A**) Representative AlphaFold 3 predicted models of NbNRC2 as a tetramer, pentamer, hexamer, heptamer, and octamer. (**B**) Superimposition of two adjacent protomers from AlphaFold 3 model on the cryo-EM model with relative positioning of the α1-helix in the CC domain. (**C**) Top view of the NbNRC2 hexamer resistosome pore with predicted α1-helices from AlphaFold 3.

Fig. S8

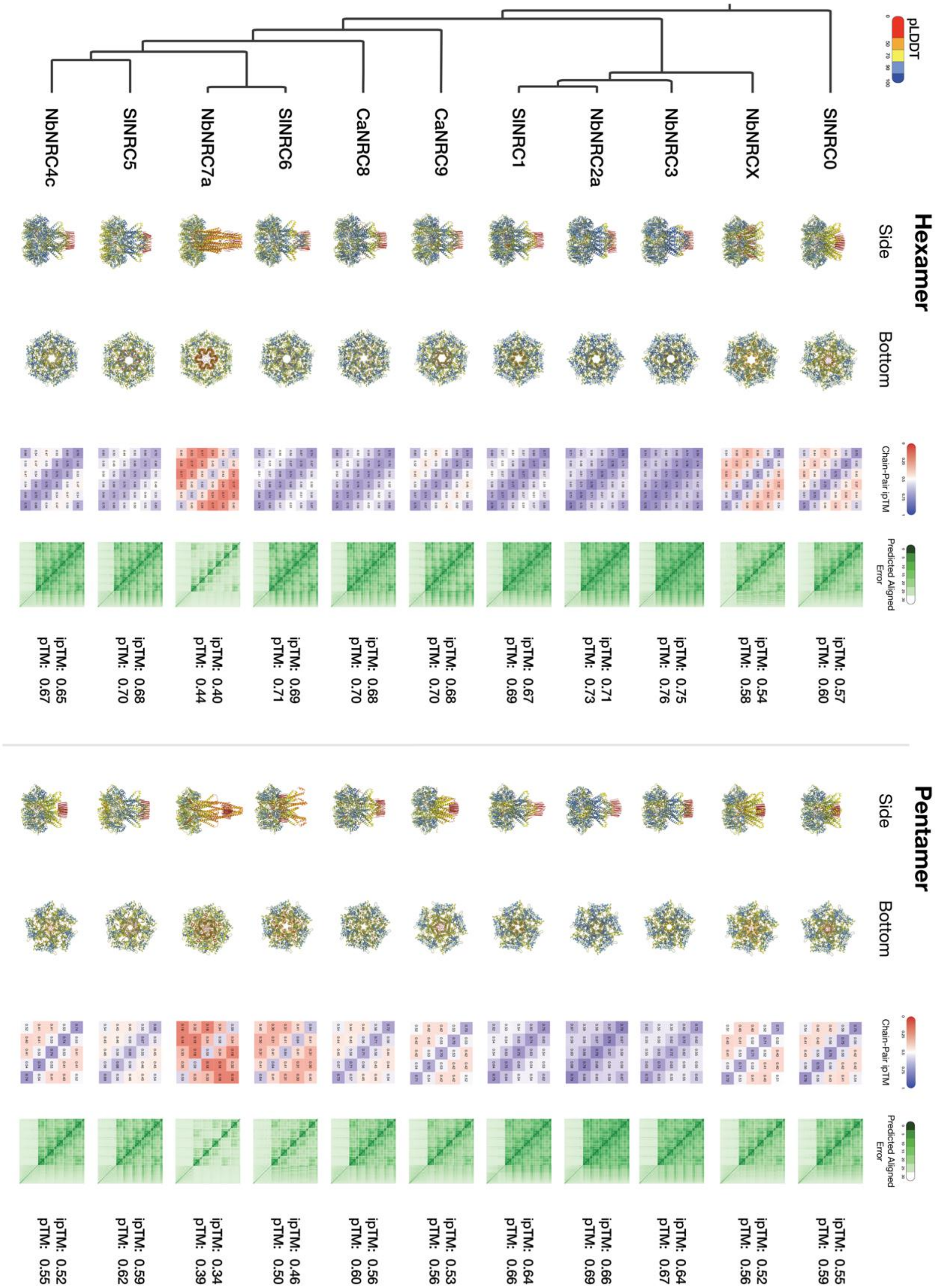

Fig. S8. Predicted pentamer and hexamer AlphaFold 3 models from representatives from NRC helper clades. Structures are colored by pLDDT values. pTM, ipTM, Chain-Pair ipTM, and predicted aligned error metrics are provided. Phylogenetic tree adapted from Selvaraj, et al., 2023 (*24*).

Fig. S9

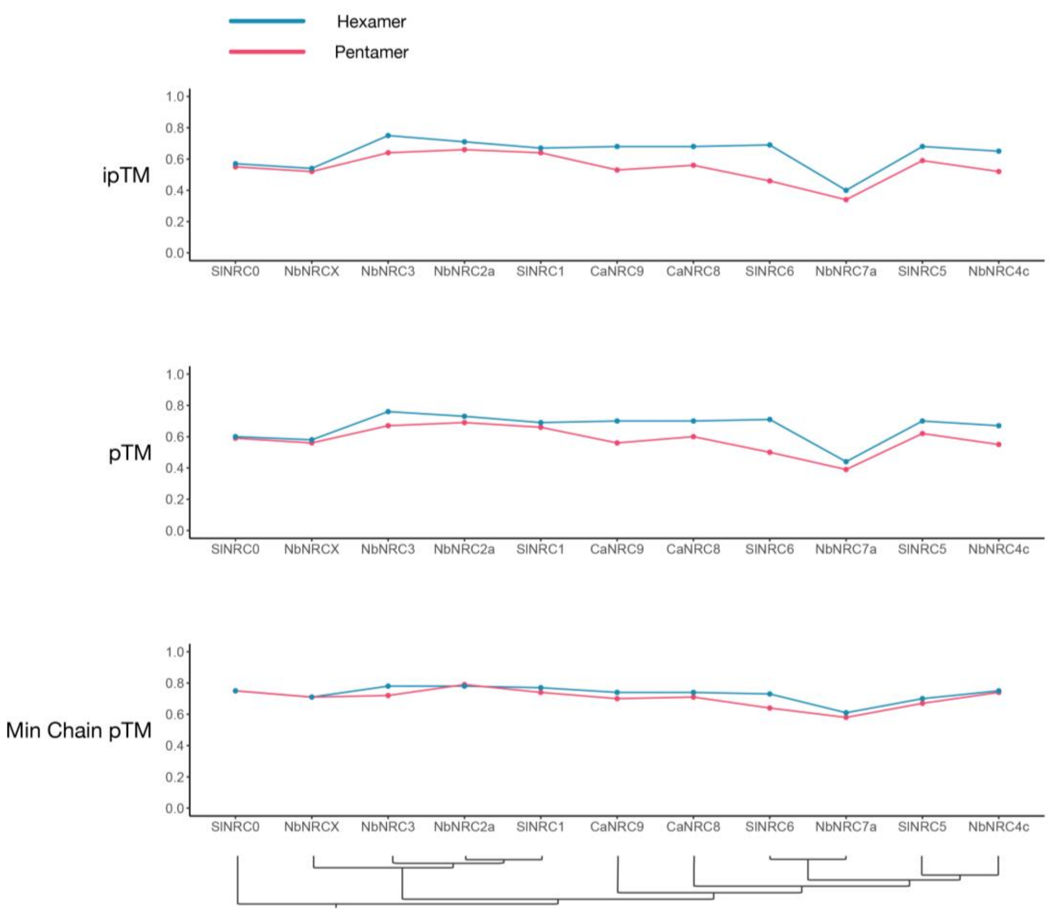

Fig. S9. ipTM, pTM, and Minimum Chain pTM confidence metrics for modelled representatives from NRC helper clades.

Fig. S10

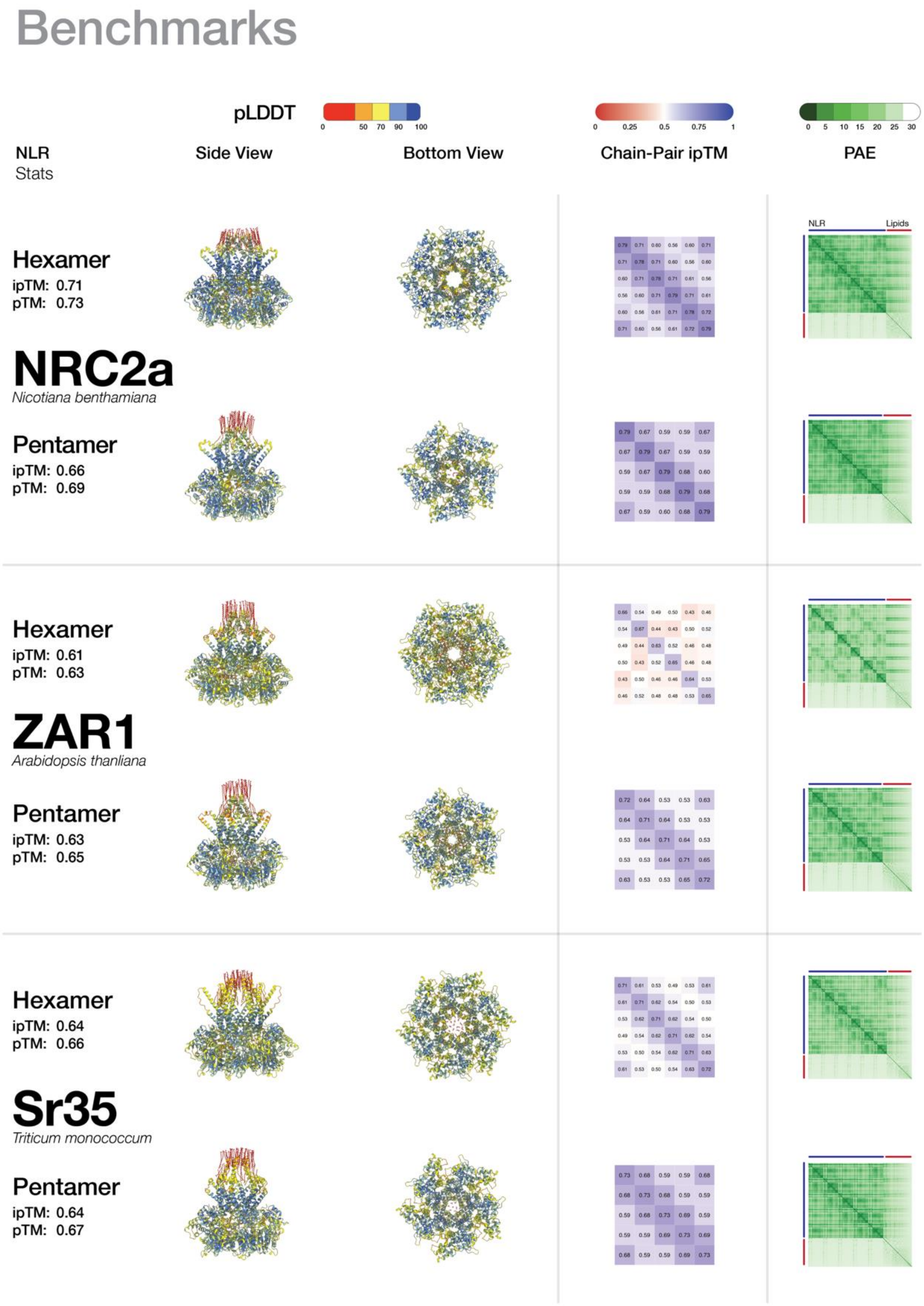

Fig. S10. AlphaFold 3 models of NbNRC2, AtZAR1, and TmSr35 as pentamers and hexamers. Structures are colored by pLDDT values. pTM, ipTM, Chain-Pair ipTM, and predicted aligned error metrics are provided.

Fig. S11

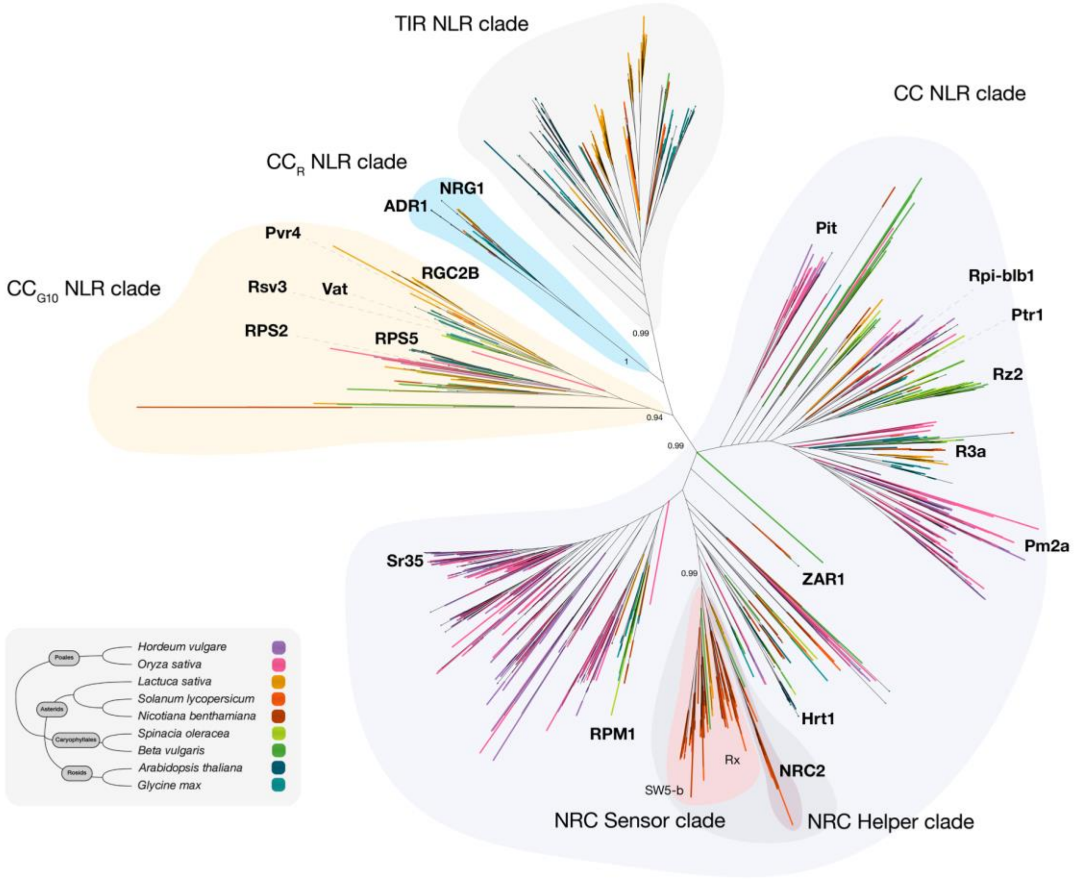

Fig. S11. Phylogenetic tree of NLRs from nine representative species from Poales, Asterids, Caryophyllales, and Rosids. Modelled NLRs are highlighted in bold on the tree. Phylogenetic tree was adapted from Contreras, et al., 2023 (*3*).

Fig. S12

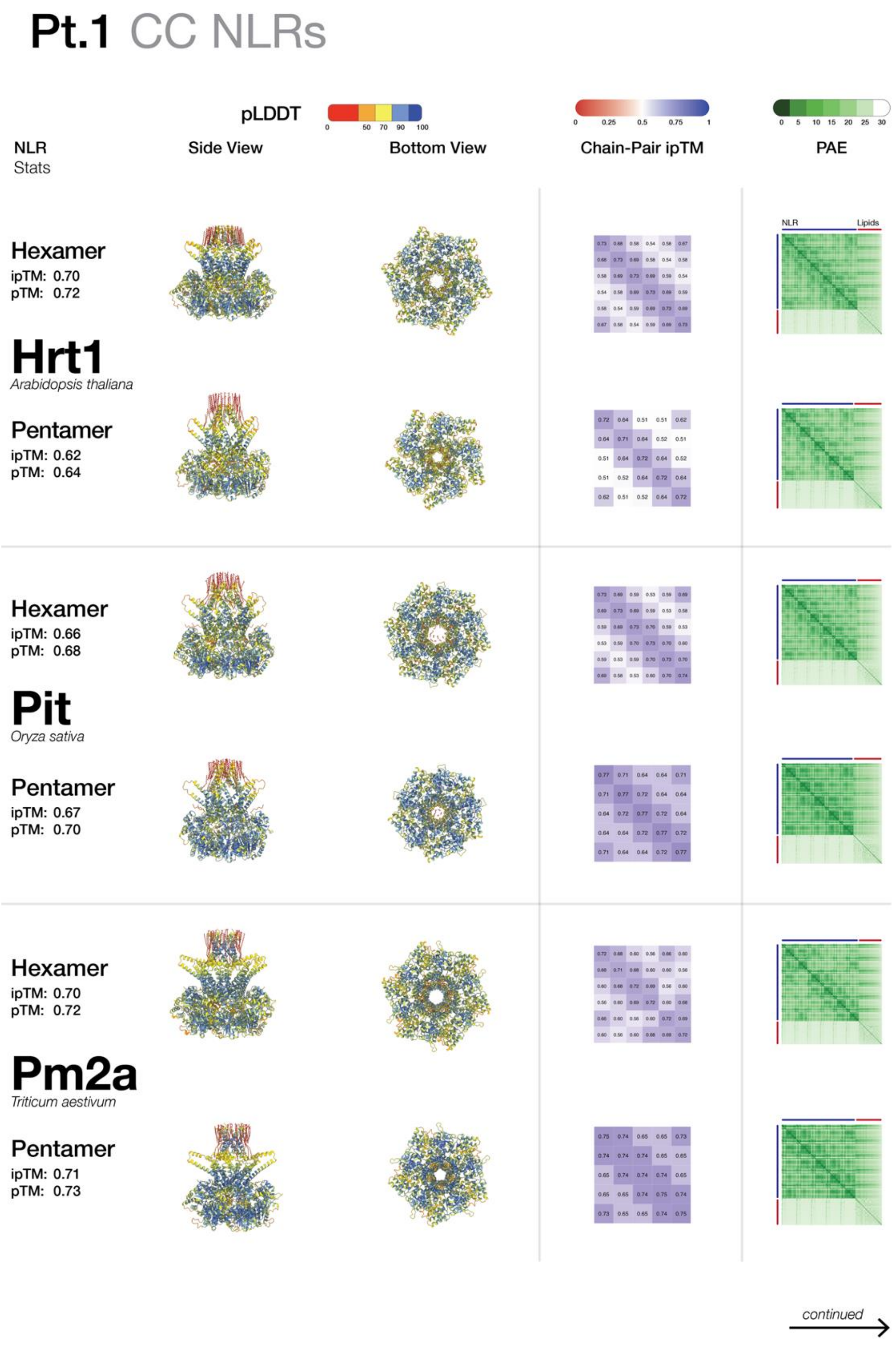

Fig. S12. AlphaFold 3 models of selected CC-NLRs, AtHrt1, PsPit, and TaPm2a, as pentamers and hexamers. Structures are colored by pLDDT values. pTM, ipTM, Chain-Pair ipTM, and predicted aligned error metrics are provided.

Fig. S13

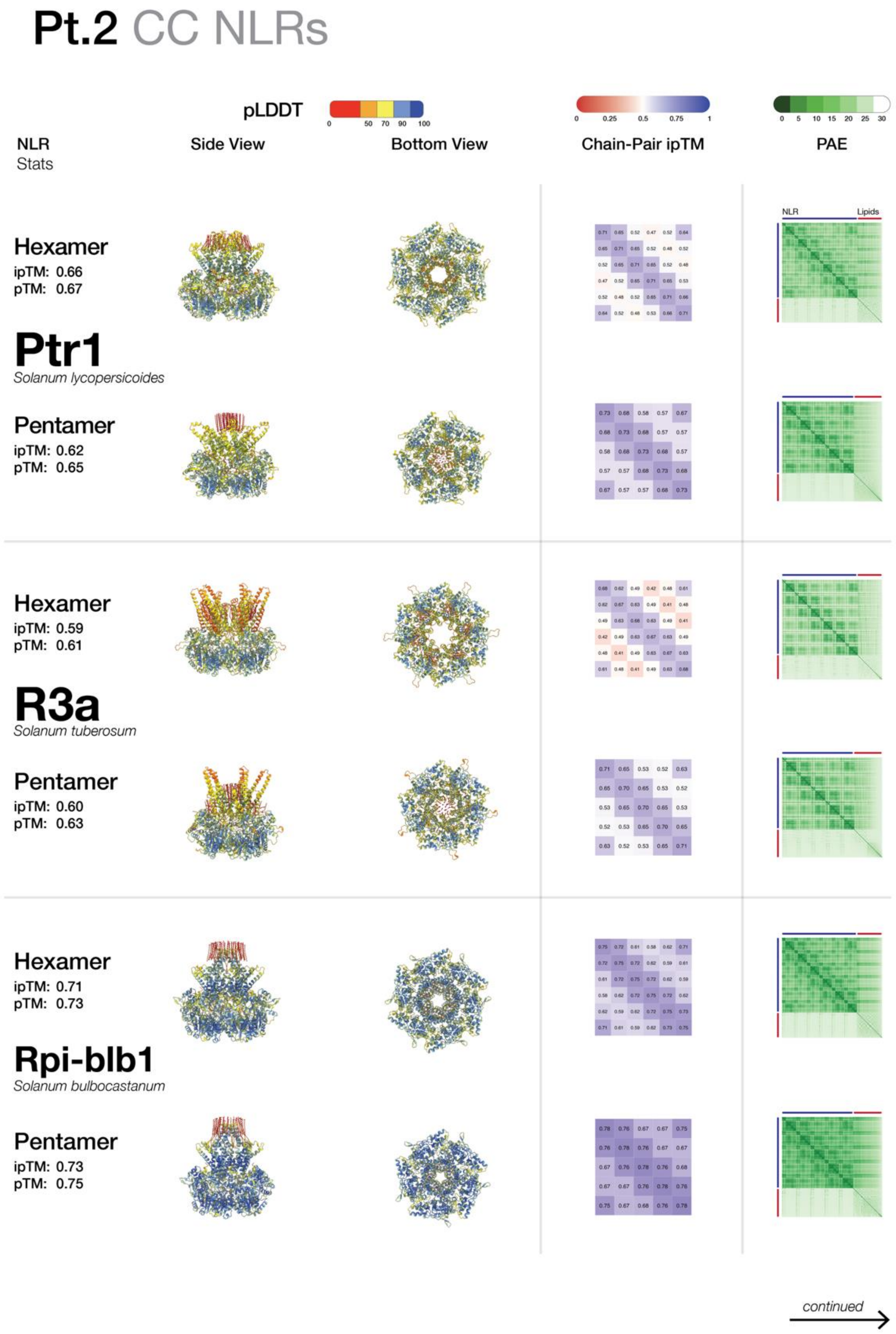

Fig. S13. AlphaFold 3 models of selected CC-NLRs, SlPtr1, StR3a, and SbRpi-blb1, as pentamers and hexamers. Structures are colored by pLDDT values. pTM, ipTM, Chain-Pair ipTM, and predicted aligned error metrics are provided.

Fig. S14

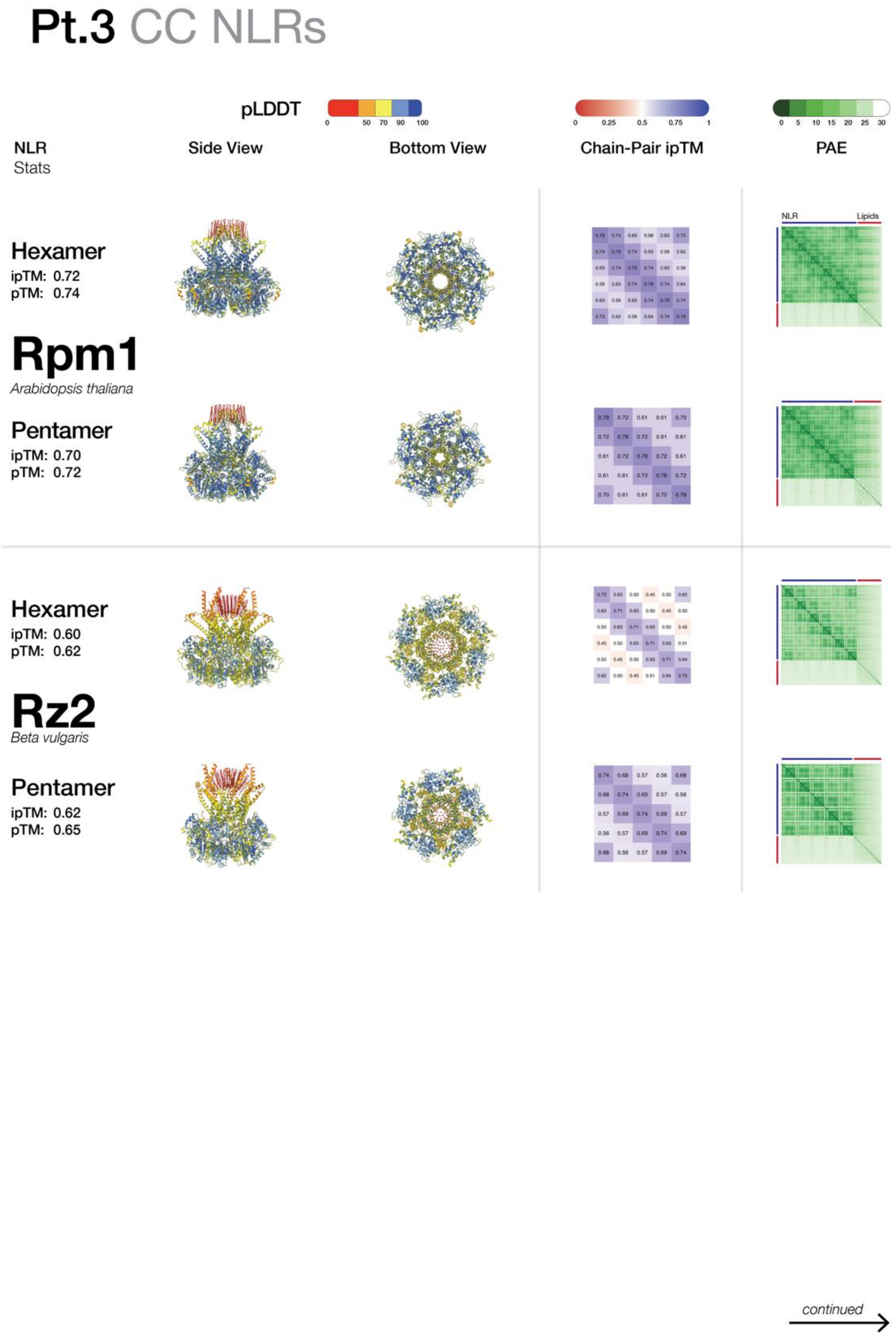

Fig. S14. AlphaFold 3 models of selected CC-NLRs, AtRpm1, and BvRz2, as pentamers and hexamers. Structures are colored by pLDDT values. pTM, ipTM, Chain-Pair ipTM, and predicted aligned error metrics are provided.

Fig. S15

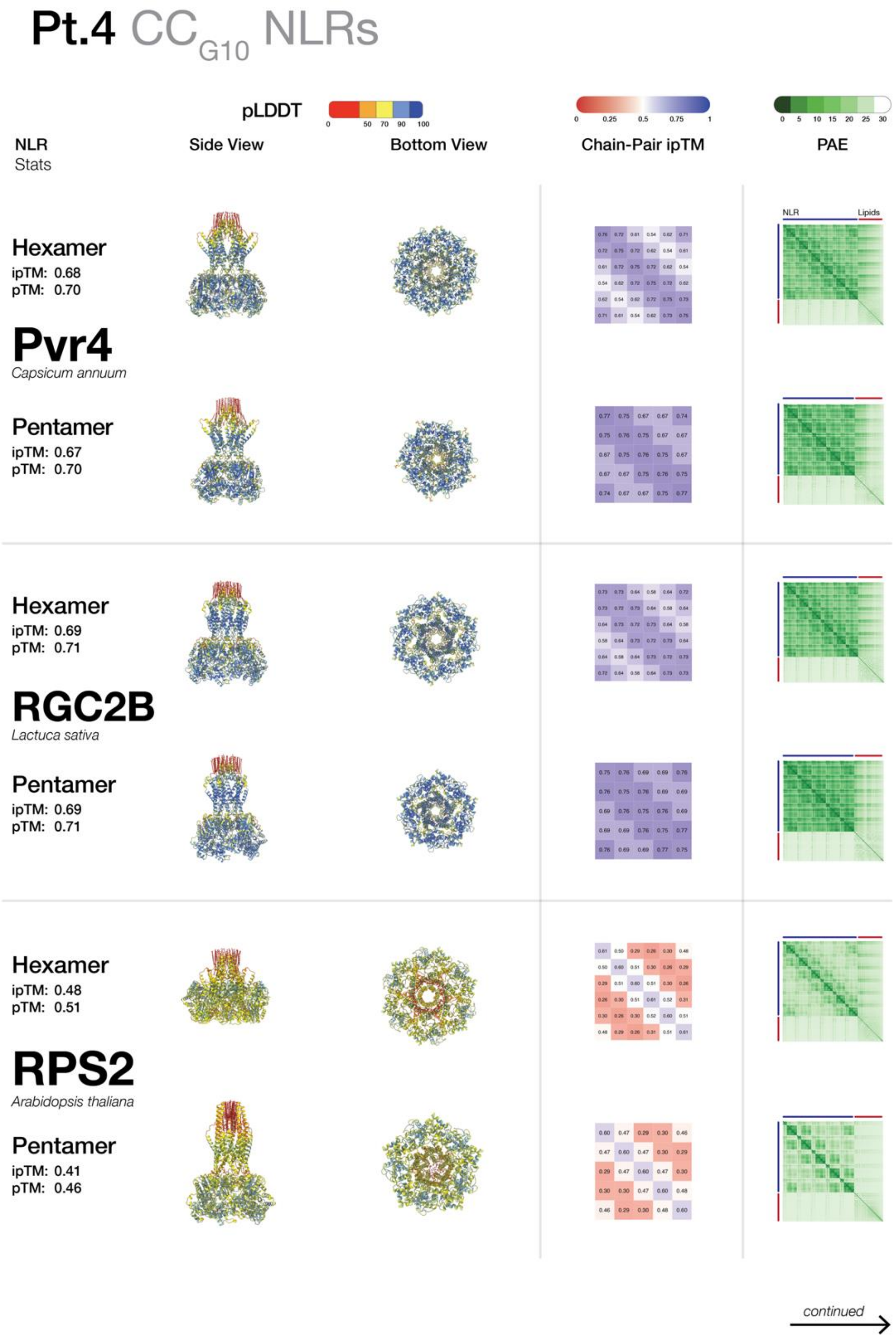

Fig. S15. AlphaFold 3 models of selected CC_{G10}-NLRs, CaPvr4, LsRGC2B, and AtRPS2, as pentamers and hexamers. Structures are colored by pLDDT values. pTM, ipTM, Chain-Pair ipTM, and predicted aligned error metrics are provided.

Fig. S16

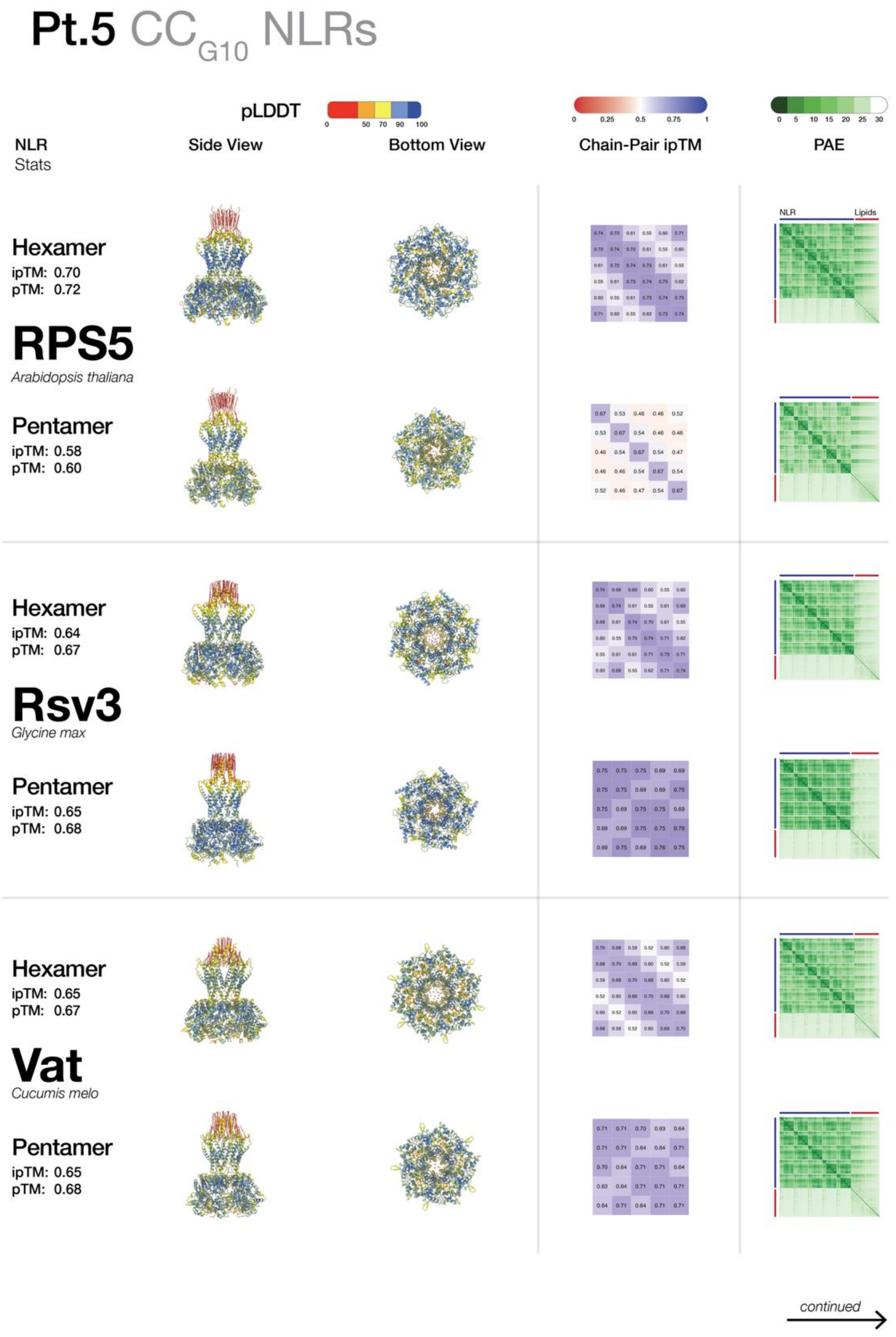

Fig. S16. AlphaFold 3 models of selected CC_{G10}-NLRs, AtRPS5, GmRsv3, and CmVat, as pentamers and hexamers. Structures are colored by pLDDT values. pTM, ipTM, Chain-Pair ipTM, and predicted aligned error metrics are provided.

Fig. S17

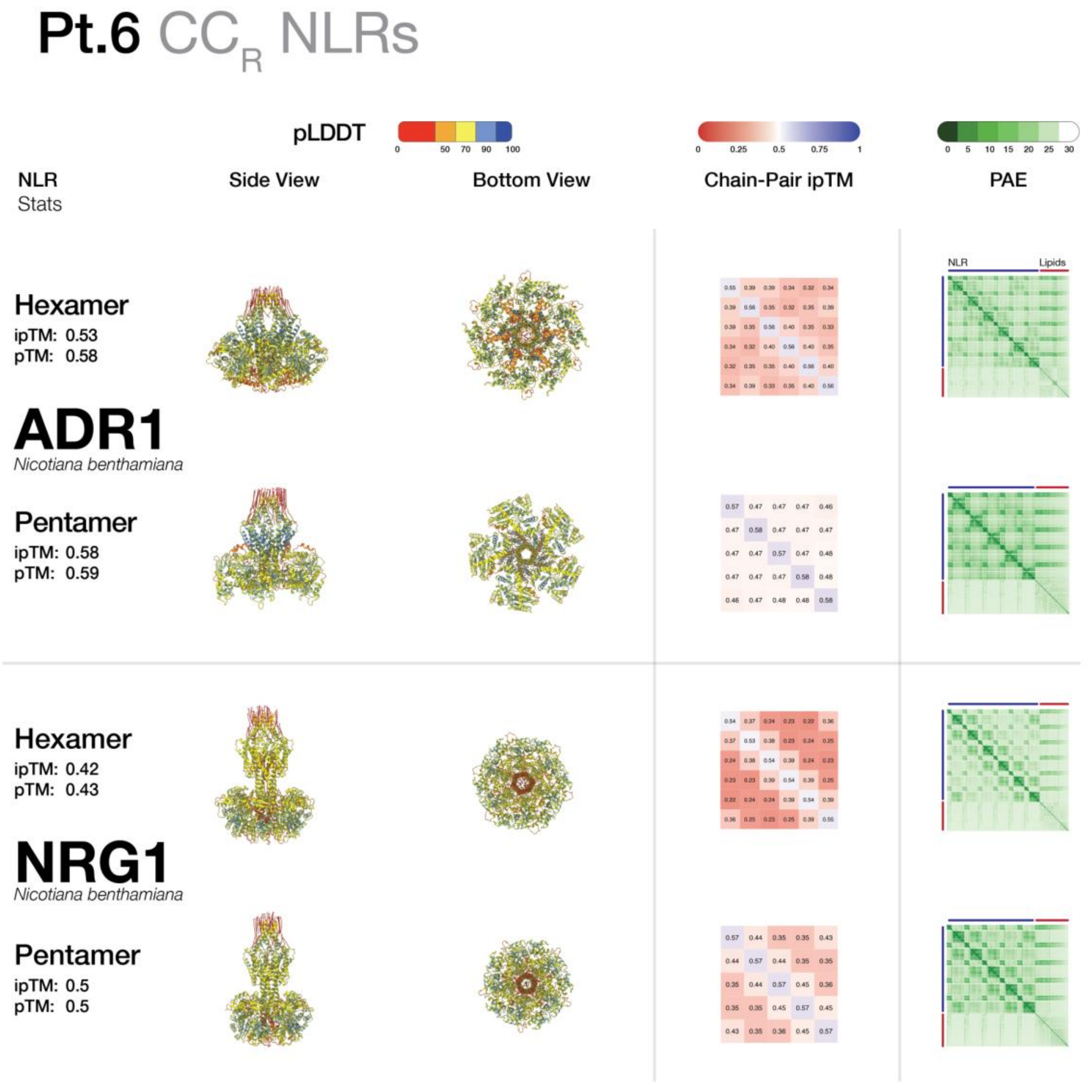

Fig. S17. AlphaFold 3 models of selected CC_R-NLRs, NbADR1, and NbNRG1, as pentamers and hexamers. Structures are colored by pLDDT values. pTM, ipTM, Chain-Pair ipTM, and predicted aligned error metrics are provided.

Fig. S18

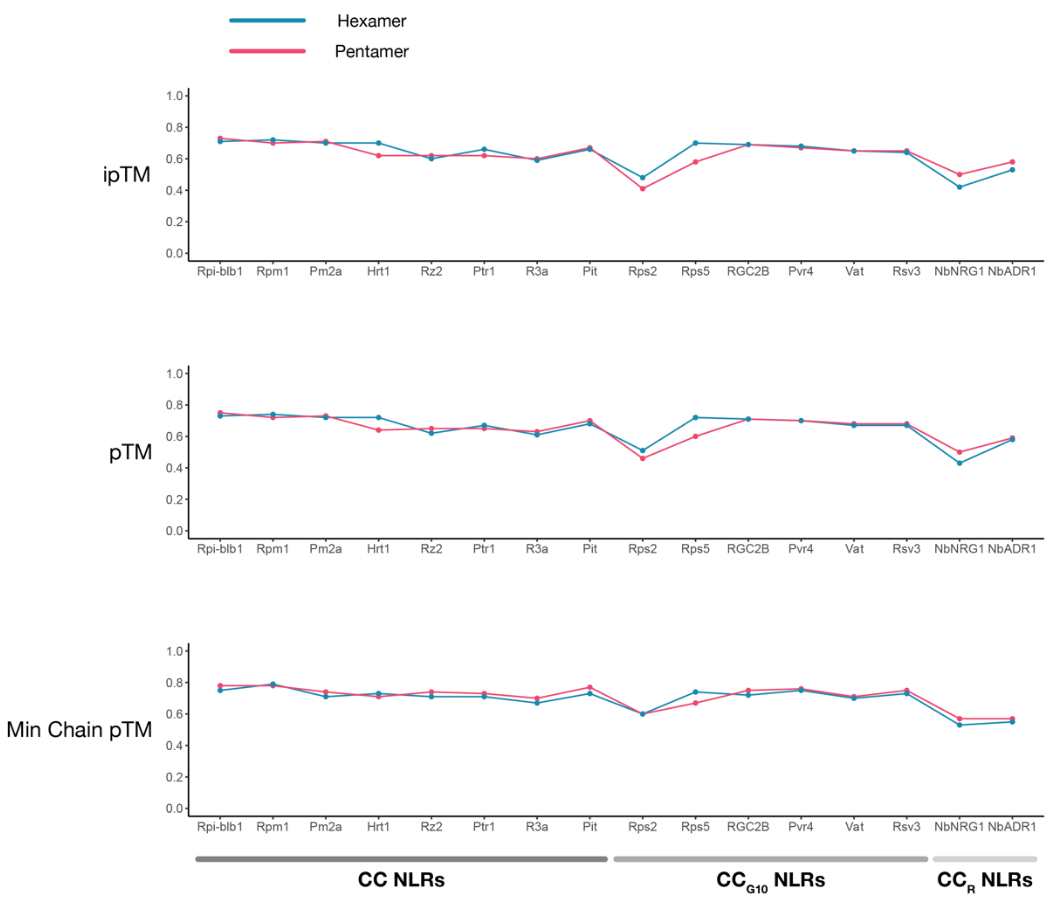

Fig. S18: ipTM, pTM, and Minimum Chain pTM confidence metrics for modelled representative CC, CC$_{G10}$, and CC$_R$ NLRs.

Table S1: Cryo-EM data collection, refinement, and validation statistics.

**Data collection**	
Pixel size (Å)	0.828
Electron flux (e^-/Å^2/sec)	25.4
Micrograph exposure time (sec)	1.95
Electron fluence per frame (e^-/Å^2)	49.5
Number of frames	50
Total electron fluence (e^-/Å^2)	25.4
Defocus range (µm)	0.6 - 4.0
Number of micrographs collected	12 908
**Cryo-EM reconstruction**	
Particles in final reconstruction	205 897
Extracted particle box size (pixels)	480
Resolution (corrected, FSC 0.143)	2.9
Sharpening B-factor (Å^2)	-109.4
**Model composition**	
Non-hydrogen atoms	35 592
Amino acid residues	4 392
Other ligands (ATP)	6
**Model statistics and validation**	
Model deposition code	9FP6
Map-to-model FSC of 0.5 (Å)	3.0
Bond length RMS deviations (Å)	0.002
Angle RMS deviations (°)	0.452
MolProbity score	1.51
Clash score, all atoms	6.19
Ramachandran favoured %	97.8
Ramachandran outlier %	0.0
Rotamer outlier %	1.4
Cβ outliers %	0.0

Movie S1: Conformational changes undergone by NbNRC2 protomers transitioning from the resting state homodimer form to the activated homohexamer.

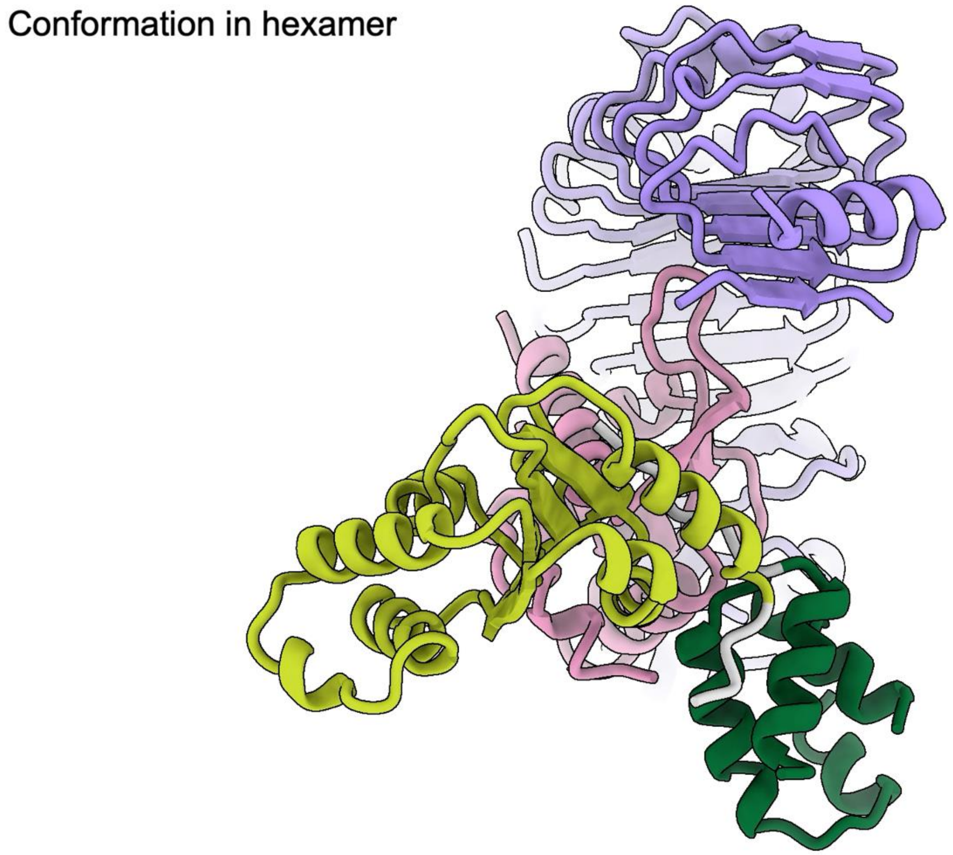

Data S1: List of modelled NLRs with their respective metadata and model statistics.

Data S2: List of contact residues from the cryo-EM model and AlphaFold 3 predicted structure.

Please refer to the published datasets here:

Toghani, A., Bozkurt, T. O., & Kamoun, S. (2024). AlphaFold 3 predicted NLR resistosome structures [Data set]. Zenodo. https://doi.org/10.5281/zenodo.11546022 *(49)*.
